# Use, satisfaction, and preference of online health services among older adults with multimorbidity in Hong Kong primary care during COVID-19

**DOI:** 10.1186/s12877-023-04061-3

**Published:** 2023-06-15

**Authors:** Zijun Xu, Dexing Zhang, Xiaoxiang Zheng, Rym C.M. Lee, Samuel Y.S. Wong, Carmen K.M. Wong

**Affiliations:** JC School of Public Health and Primary Care, The Chinese University of Hong Kong, Hong Kong, China

**Keywords:** COVID-19, Multimorbidity, Online health service, Primary care

## Abstract

**Background:**

The use of online and mobile internet and social media has been increasing in healthcare service delivery. However, there is limited literature on the acceptance and use of online health services for older adults with multimorbidity who require more medical care and assistance. This study aims to explore the use of social media in older adults with multimorbidity in Hong Kong primary care and to assess the feasibility and usage of online health services in this population, including satisfaction, preference, and problems encountered.

**Methods:**

This is a cross-sectional study among older adults with multimorbidity conducted between November 2020 and March 2021 in a Hong Kong primary care programme. Online and face-to-face services were offered based on the needs of the participants. Demographic characteristics and health conditions were assessed at baseline. Participants using online services were invited to complete a feedback questionnaire.

**Results:**

The study included 752 participants, of which 66.1% use social media every day. Participants who declined to use online services were found to be significantly older, live alone, have lower income, have social security assistance, have greater cognitive decline, and be less depressed (p < 0.05). Non-responders to the online questionnaire had fewer years of education and greater cognitive decline (p < 0.05). The median satisfaction with the online services was 8 (interquartile range: 7, 9), and 14.6% of the participants preferred online more than face-to-face services. Lower education levels, fewer internet connection issues, and more self-efficacy on mobile apps were associated with a higher level of online satisfaction after adjustment (p < 0.05). Fewer internet connection issues and more self-efficacy on mobile apps were associated with participants’ preference for online services (p < 0.05).

**Conclusions:**

More than half of Hong Kong older adults with multimorbidity in primary care use social media daily. Internet connection issues can be a significant barrier to the usage of online services in this population. Prior use and training can be beneficial to enhance use and satisfaction in older adults.

## Introduction

The aging population is a global challenge and will be an increasing challenge for years to come [1]. In 2019, about 9% of the world’s population was aged 65 years old or above, and this will increase to 16% by 2050 [1]. Hong Kong, the city with the longest life expectancy of 84.7 years old, have 35.7% of the population aged 55 years old or above and 19.1% aged 65 or above in 2020 [2, 3]. In older adults aged 60 and above in Hong Kong, at least half of them have multimorbidity [4]. According to government statistics released in 2020, the penetration rate of smartphones among people over 65 years old in Hong Kong increased from 6.9% to 2012 to 65.1% in 2019 [5]. Another study also indicated that the rate among people over 65 years old in Hong Kong in 2020 was 73.8% in males and 68.1% in females [6]. With the popularity of smartphones, Hong Kong has a relatively high smartphone penetration rate among older adults, and the prevalence increased year by year. There is a growing need to utilize smartphone use in older adults with multimorbidity to enhance their health.

Social media refers to collaborative platforms that can be used to create virtual communities, such as blogs, Facebook, Twitter, and WhatsApp [7–9]. In the past few years, there has been increasing use of the internet and social media applications in healthcare service delivery [10]. Due to COVID-19, many services were also converted from face-to-face mode to online service, which can be delivered by audio or video call [11]. Online health services have been shown to reduce healthcare costs, reduce patient travel and wait times, improve efficiency, and can provide care and resources to patients having limited access to healthcare [12]. The scope of online health services has extended to different diseases, conditions, and populations, including college students [13], children and adolescents [14, 15], people with mental health problems [16, 17], other chronic conditions such as hypertension and obesity [18, 19], and high-risk behaviors such as smoking [20]. Online health services can help people to manage their physical, mental, and social well-being, as well as to prevent diseases [21]. This can be of greater benefit to older adults who suffer from multiple conditions with aging, which impact their physical, social and mental health.

The acceptance of online services for older adults may be different from that of the younger generation as they can less proficient in using smart electronic products. Studies conducted to assess the gaps in online health services among older adults show low participation rates, high drop-out rates, and low efficacy as common problems when using online health services [22]. Perceived benefits of services, timing of programme introduction, and competency and confidence with digital technology are factors associated with initial online health service uptake and engagement [23]. In addition, other contributors to engagement include participants’ re-evaluation of the ongoing benefits, support and peer networks, behaviour change techniques (e.g., setting individual goals and giving feedback), and novelty factors of the service [23].

Meanwhile, there remains a lack of high-quality evidence on whether online health services can improve the physiological, psychological, and social health of older adults [24]. The use of social media can have additional benefits for older adults beyond content provision and organization and can be helpful in enhancing the social support environment and deeper understanding [9]. The Technology Acceptance Model (TAM) links social media use, user’s satisfaction, and learner’s performance [7] and shows how perceived usefulness, perceived ease of use and behavioural intentions can impact actual use.

This study aims to explore the use of social media use in older adults with multimorbidity in Hong Kong primary care and the feasibility and usage of online health services including satisfaction, preference, and problems encountered.

## Methods

### Study design and participants

This is a cross-sectional study conducted in a primary care programme with online and face-to-face services in Hong Kong. The study was performed in accordance with the Declaration of Helsinki. Ethical approval was obtained from the Joint Chinese University of Hong Kong - New Territories East Cluster Clinical Research Ethics Committee (CREC2019.329). All participants provided written informed consent.

Eligible participants) were 55 years old or above, 2) had at least one physical need (e.g., chronic pain, sarcopenia) with at least one mental or social need (e.g., depression, cognitive impairment, loneliness), or lived alone or only lived with another older adult. Details were presented in “Criteria for entering each service” below. The exclusion criteria included (1) seeing a psychologist in the past six months, (2) having psychosis or bipolar disorder, (3) accessing substance abuse services, and (4) being actively suicidal.

Primary care patients included in this study were recruited from general outpatient clinics (GOPCs) in the New Territory East Cluster (NTEC) in Hong Kong from November 2019 to September 2020. The trained research assistants did the initial screening in GOPCs, and if eligible, the participants were further assessed by the study nurse. The study was conducted in a university-affiliated primary care clinic. Participants were offered ten different services, including physical, social, or mental activities, for at least four weeks. Each service was conducted for four sessions, and each session was held for around one hour. Participants who attended the 4-week regular sessions can attend reunion activities a maximum of twice a month. The services were provided by trained nurses, social workers, research assistants, and exercise coaches.

Due to the COVID-19 epidemic and the consequent ban on group gatherings directed by the Hong Kong government, the research team switched face-to-face sessions to online sessions via an online platform called Zoom on 20 April 2020. No face-to-face sessions were offered during this period. The participants were trained on how to use Zoom personally or through phone calls. The research team also uploaded the training video to YouTube and Facebook to introduce Zoom at the same time. All the zoom links, workshop notes and supplementary notes were sent via WhatsApp. The participants who attended the online sessions from 20 to 2020 to 9 October 2020 were contacted via Whatsapp from 17 to 2020 to 11 March 2021 to complete an online feedback questionnaire regarding their experience of the online services. One phone reminder was made by the research team to participants who did not reply to Whatsapp.

### Criteria for entering each service

Participants with different conditions were allocated to different services. Services included workshops for anxiety, depression, cognitive training, loneliness, sarcopenia and frailty, social support, mindfulness, and pain, disease, and drug management. The criteria for allocation were as follows: anxiety: score was five or more 7-item Generalized Anxiety Disorder (GAD-7) [25]; depression: score was ≥ 5 on the 9-item Patient Health Questionnaire (PHQ-9) [26]; cognitive training: score less than 22 on Hong Kong Montreal Cognitive Assessment (HK-MoCA) [27]; loneliness: score of 3 or more in the 6-item De Jong Gierveld Loneliness Scale [28]; social support workshop if the score of Multidimensional Scale of Perceived Social Support (MSPSS) [29] was ≤ 5; allocated to pain management if the participants had pain lasting at least three months in the past year; allocated to the session for sarcopenia and frailty if the participants had a SARC-F [30] score ≥ 4 or a FRAIL [31] score ≥ 1. If the participants had suboptimal diabetes mellitus (DM) control with the latest HbA1c ≥ 7 or suboptimal hypertension (HT) control with blood pressure ≥ 140/90, DM/HT management was offered. Drug management was offered to the participants if they had polypharmacy with five or more long-term drugs or had poor drug compliance. The remaining participants, who had less than two conditions above and lived alone or lived with another older adult, can join social support workshops or cognitive training. Mindfulness was offered only as one of the reunion sessions after they finished the sessions for depression, anxiety, and loneliness.

### Measurements

#### Baseline assessments

Demographic characteristics measured included age, years of education, marital status, type of housing, caregiver, family income, social media use, social security assistance, and regular medications. Baseline cognition, depressive symptoms, social support, and loneliness were measured by HK-MoCA [27], PHQ-9 [26], MSPSS [29], and the 6-item De Jong Gierveld Loneliness Scale [28], respectively. The assessments were conducted by trained nurses, social workers, and research assistants through face-to-face interviews.

#### Online intervention feedback assessment

Satisfaction and preference for the online service were measured as the outcomes. Overall satisfaction with the online service was measured on a 10-point Likert scale from 1 (very unsatisfied) to 10 (very satisfied), with a higher score indicating higher satisfaction. Preference was dichotomized as preferring online and preferring face-to-face intervention.

Issues with internet connection were measured on a Likert scale and categorized as never, seldom, sometimes, often, and always. The use of social media in the past two weeks, as well as its usage and frequency, were recorded. Self-efficacy ranged from 1 to 10, with a higher score indicating higher self-efficacy on mobile apps. Assistance needed on using mobile apps was categorized as never, sometimes, often, and always. Other information included the number of online sessions attended, other online learning experiences, new user to the Zoom platform, having a quiet and suitable environment and devices used to attend online services. Two open questions were asked about their opinion on online health services.

### Statistical analysis

Data were presented as the median and interquartile range (IQR) for continuous variables and numbers and percentages for categorical variables. To compare the differences between participants who attended and who were not willing to use online services, as well as participants who were willing and unwilling to give feedback on online services, the independent sample t-test was used for continuous variables and Pearson’s Chi-Square test for categorical variables. Univariable and multivariable ordinal logistic regressions were performed to assess the factors associated with online intervention satisfaction and preference. A p-value less than 0.05 (two-tailed) was considered statistically significant. All statistical analyses were performed using Stata version 16.

## Results

In the first year of the programme till September 2020, 752 participants with multimorbidity were recruited in the primary care programme. The median age was 69 (IQR: 65, 73) years old. Among these participants, 372 (49.5%) used social media more than three times a day, 125 (16.6%) used 1–3 times a day, 53 (7.1%) used once a week, 24 (3.2%) used several times per month, and 178 (23.7%) used less than once a month.

A total of 624 sessions were offered during this period, and 4739 attendances were recorded, among which 272 sessions were conducted online with 2109 attendances. The usage of the ten different services offered to the participants in the first year of the programme is summarized in Table 1.

**Table 1:** The usage of the services offered in the first year of the primary care programme

Type of service	Total			Online		
	Sessions	Participants	Attendances	Sessions	Participants	Attendances
Pain management	102	223	932	53	135	460
Session for sarcopenia and frailty	96	237	873	54	142	434
Social support	88	180	692	-	-	-
Session for anxiety	64	206	585	39	160	329
Session for loneliness	64	107	348	29	62	137
Session for depression	60	144	448	35	95	213
Cognitive training	60	53	166	-	-	-
Mindfulness	36	74	346	27	71	311
DMHT management	28	61	221	22	52	162
Drug management	26	46	128	13	28	63

DMHT: diabetes mellitus and hypertension

Among all the participants, 429 were offered online services and 362 (84.4%) of them attended at least one session of the online service. Compared with those using online services, participants who declined to use online services were found to be older (median: 71 vs. 68 years old, p < 0.001), live alone (32.8% vs. 20.4%, p = 0.025), have lower income ( ≥10,000 HKD, 17.9% vs. 30.9%, p = 0.031), have more social security assistance (61.2% vs. 29.6%, p = 0.001), have greater cognitive decline(median MoCA: 25 vs. 26, p < 0.001), and less depressed (median PHQ-9: 4 vs. 6, p = 0.006)(Table 2).

**Table 2:** Differences between participants who used and declined to use online services

Characteristics	Participants used online services (n = 362)#	Participants declined to use online services (n = 67)#	p^
Age (years)	68 (65, 72)	71 (66.5, 75)	< 0.001*
Education year	8.5 (6, 11)	6 (6, 9)	0.071
Marriage (married)	231 (63.8)	37 (55.2)	0.182
Type of housing			0.566
Public rental housing	154 (42.5)	34 (50.7)	
Homeownership scheme flats	82 (22.7)	13 (19.4)	
Private housing	105 (29.0)	15 (22.4)	
Village housing	16 (4.4)	3 (4.5)	
Living alone	74 (20.4)	22 (32.8)	0.025*
Living with partner	221 (61.0)	36 (53.7)	0.250
Living with children	148 (40.9)	22 (32.8)	0.210
Income ( ≥10,000 HKD)	112 (30.9)	12 (17.9)	0.031*
Social media use every day	253 (69.9)	41 (61.2)	0.159
Having social security assistance	107 (29.6)	41 (61.2)	0.001*
Number of regular medications	2 (1, 4)	2 (1, 4)	0.837
MoCA	26 (24, 28)	25 (21, 28)	< 0.001*
PHQ-9	6 (3, 10)	4 (2,7)	0.006*
MSPSS	4.3 (2.8, 5.4)	4.6 (2.8, 5.8)	0.577
Loneliness	4 (2, 5)	3 (1, 5)	0.445

Loneliness: 6-item De Jong Gierveld Loneliness Scale; MoCA: Montreal Cognitive Assessment 5-Minute Protocol Hong Kong Version; MSPSS: Multidimensional Scale of Perceived Social Support; PHQ-9: Patient Health Questionnaire-9

#The results were presented as median (IQR) or n (%). ^Independent sample t-test for continuous variables and Pearson’s Chi-Square test for categorical variables were used for data analysis. *p < 0.05

Of the 362 participants who used online services and were invited to complete a questionnaire regarding their feedback on their online experiences, 213 participants responded to the questionnaire with a response rate of 58.8%. Their median satisfaction with the online services was 8 (interquartile range: 7, 9) out of a total score of 10 (Table 3). Only 31 (14.6%) of the participants with multimorbidity preferred online service more than face-to-face service (Table 3). Most of the responders (n = 206, 96.7%) used Whatsapp, and 174 (81.7%) used Whatsapp most often. Most of them (n = 193, 90.6%) used smartphones to use online services, and their use of Zoom platform was mostly taught by non-governmental organizations (n = 123, 57.7%). Compared with the 213 participants, the 149 participants who did not respond to the questionnaire had fewer years of education (7 vs. 9, p < 0.001) and had greater cognitive decline (MoCA, 26 vs. 27, p < 0.001) (Table 4).

**Table 3:** Social media use and online learning of the participants who responded to the survey (n = 213)

Outcomes	Median (IQR) / n (%)
**Social media use**	
Using Whatsapp	206 (96.7)
Using Whatsapp most often#	174 (81.7)
**Online learning**	
Satisfaction (1–10)	8 (7, 9)
Preference on online learning	31 (14.6)
Device used to use service	
Smart phone	193 (90.6)
Tablet computer	35 (16.4)
Computer	15 (7.0)
Who taught them to use Zoom	
Themselves	22 (10.3)
Family member or friends	69 (32.4)
NGO	123 (57.7)

# When compared with Facebook, Twitter, Instagram, Facetime, Zoom, WeChat, and Blog. NGO: non-governmental organizations

**Table 4:** Differences between participants who responded and not responded to the online feedback questionnaire

Characteristics	Participants responded to the online feedback questionnaire (n = 213)#	Participants did not respond to the online feedback questionnaire (n = 149)#	p^
Age (years)	68 (65, 71)	68 (65, 72)	0.137
Education year	9 (6, 11)	7 (6, 10)	< 0.001*
Marriage (married)	131 (61.5)	100 (67.1)	0.274
Type of housing			0.581
Public rental housing	88 (41.3)	66 (44.3)	
Homeownership scheme flats	49 (23.0)	33 (22.1)	
Private housing	61 (28.6)	44 (29.5)	
Village housing	12 (5.6)	4 (2.7)	
Living alone	48 (22.5)	26 (17.4)	0.238
Living with partner	127 (59.6)	94 (63.1)	0.456
Living with children	90 (42.3)	58 (38.9)	0.560
Income ( > = 10,000 HKD)	64 (30.0)	48 (32.2)	0.661
Social media use every day	157 (73.7)	96 (64.4)	0.058
Having social security assistance	61 (28.6)	46 (30.9)	0.362
Number of regular medications	2 (1, 4)	2 (1, 4)	0.060
MoCA	27 (25, 28)	26 (23, 28)	< 0.001*
PHQ-9	6 (3, 10)	7 (4, 11)	0.066
MSPSS	4.3 (3.0, 5.4)	4.1 (2.7, 5.3)	0.317
Loneliness	4 (2, 5)	3 (2, 5)	0.520

Loneliness: 6-item De Jong Gierveld Loneliness Scale; MoCA: Montreal Cognitive Assessment 5-Minute Protocol Hong Kong Version; MSPSS: Multidimensional Scale of Perceived Social Support; NGO: non-governmental organization; PHQ-9: Patient Health Questionnaire-9

*p < 0.05. #The results were presented as median (IQR) or n (%). ^Independent sample t-test for continuous variables and Pearson’s Chi-Square test for categorical variables were used for data

analysis

Tables 5 and 6 summarized the results of the ordinal logistic regression exploring the factors associated with participants’ satisfaction and preference. In multivariable regression, higher education (OR = 0.65, 95% CI = 0.48–0.89) and internet connection issues (OR = 0.50, 95% CI = 0.37–0.66) were negatively associated with satisfaction with the online services, and higher self-efficacy on mobile apps (OR = 1.58, 95% CI = 1.33–1.88) was associated with greater online satisfaction after adjustment. The results also indicated that after adjustment, participants facing internet connection issues preferred online services less (OR = 0.52, 95% CI = 0.31–0.87), and participants with higher self-efficacy on mobile apps preferred online services more (OR = 1.41, 95% CI = 1.05–1.90).

**Table 5:** Factors associated with satisfaction on online intervention using ordinal logistic regression (n = 213)

Variables	Satisfaction			
	Crude OR (95% CI)	p	Adjusted OR (95% CI)	p
Age	1.04 (1.00, 1.09)	0.069	1.03 (0.98, 1.08)	0.285
Higher education	0.82 (0.63, 1.06)	0.123	0.65 (0.48, 0.89)	0.006*
Number of people living with	0.94 (0.74, 1.20)	0.626	0.98 (0.74, 1.29)	0.885
Being caregiver	1.05 (0.63, 1.75)	0.844	0.78 (0.46, 1.33)	0.365
Income	0.99 (0.85, 1.16)	0.928	1.03 (0.85, 1.24)	0.791
Number of sessions attended ( > = 5)	1.14 (0.71, 1.84)	0.593	1.21 (0.71, 2.08)	0.478
Other online learning experience	1.16 (0.69, 1.96)	0.578	0.91 (0.48, 1.74)	0.782
First time using Zoom	1.23 (0.66, 2.30)	0.518	1.83 (0.83, 4.03)	0.134
Quiet and suitable environment	1.60 (0.71, 3.61)	0.258	1.02 (0.39, 2.67)	0.972
Internet connection issues	0.57 (0.44, 0.74)	< 0.001*	0.50 (0.37, 0.66)	< 0.001*
Social media use in past 2 weeks	1.43 (0.67, 3.06)	0.351	0.98 (0.36, 2.67)	0.971
Frequent social media usage	1.16 (0.93, 1.45)	0.178	0.93 (0.69, 1.25)	0.623
Self-efficacy on mobile apps	1.45 (1.26, 1.67)	< 0.001*	1.58 (1.33, 1.88)	< 0.001*
Assistance on using mobile apps	0.57 (0.38, 0.85)	0.006	0.96 (0.59, 1.56)	0.873
MoCA	0.97 (0.89, 1.06)	0.485	0.93 (0.84, 1.02)	0.131
PHQ-9	0.97 (0.92, 1.03)	0.357	1.05 (0.98, 1.13)	0.189
MSPSS	1.07 (0.92, 1.24)	0.412	1.11 (0.93, 1.34)	0.258
Loneliness	0.92 (0.81, 1.04)	0.176	0.92 (0.79, 1.08)	0.321

*p < 0.05. Assistance on using mobile apps: 1 = never, 2 = sometimes, 3 = often, 4 = always; Loneliness: 6-item De Jong Gierveld Loneliness Scale; MoCA: Montreal Cognitive Assessment 5-Minute Protocol Hong Kong Version; MSPSS: Multidimensional Scale of Perceived Social Support; Internet connection issues: 1 = never, 2 = seldom, 3 = sometimes, 4 = often, 5 = always; PHQ-9: Patient Health Questionnaire-9; Social media usage: 1 = less than once a month, 2 = several times a month, 3 = once a week, 4 = 1–3 times a day, 5 = more than 3 times a day

**Table 6:** Factors associated with preference on online intervention using ordinal logistic regression (n = 213)

Variables	Preference on online intervention#			
	Crude OR (95% CI)	p	Adjusted OR (95% CI)	p
Age	0.91 (0.84, 0.98)	0.013*	0.91 (0.83, 1.01)	0.080
Higher education	1.48 (1.01, 2.18)	0.044*	1.03 (0.62, 1.72)	0.898
Number of people living with	1.59 (1.06, 2.37)	0.023*	1.45 (0.87, 2.42)	0.155
Being caregiver	1.81 (0.83, 3.91)	0.134	2.28 (0.90, 5.75)	0.082
Income	1.26 (1.01, 1.56)	0.037*	1.21 (0.92, 1.59)	0.170
Number of sessions attended ( > = 5)	1.27 (0.59, 2.73)	0.536	1.59 (0.61, 4.14)	0.341
Other online learning experience	1.22 (0.54, 2.78)	0.630	1.23 (0.37, 4.14)	0.735
First time using Zoom	0.70 (0.28, 1.78)	0.458	0.93 (0.21, 4.03)	0.922
Quiet and suitable environment	3.7 (0.48, 28.65)	0.210	2.91 (0.3, 28.17)	0.357
Internet connection issues	0.54 (0.36, 0.80)	0.002*	0.52 (0.31, 0.87)	0.013*
Social media use in past 2 weeks	1.49 (0.42, 5.26)	0.539	0.91 (0.15, 5.35)	0.916
Frequent social media usage	1.06 (0.75, 1.50)	0.745	0.74 (0.45, 1.23)	0.251
Self-efficacy on mobile apps	1.34 (1.07, 1.68)	0.010*	1.41 (1.05, 1.90)	0.021*
Assistance on using mobile apps	0.71 (0.38, 1.32)	0.276	1.60 (0.68, 3.78)	0.282
MoCA	1.14 (0.98, 1.33)	0.095	1.00 (0.84, 1.19)	0.969
PHQ-9	0.96 (0.88, 1.05)	0.413	0.96 (0.85, 1.09)	0.559
MSPSS	0.93 (0.74, 1.17)	0.542	0.77 (0.56, 1.07)	0.120
Loneliness	1.04 (0.85, 1.27)	0.709	0.96 (0.73, 1.26)	0.748

*p < 0.05. #Preference on offline learning as the reference group

Assistance on using mobile apps: 1 = never, 2 = sometimes, 3 = often, 4 = always; Loneliness: 6-item De Jong Gierveld Loneliness Scale; MoCA: Montreal Cognitive Assessment 5-Minute Protocol Hong Kong Version; MSPSS: Multidimensional Scale of Perceived Social Support; Internet connection issues: 1 = never, 2 = seldom, 3 = sometimes, 4 = often, 5 = always; PHQ-9: Patient Health Questionnaire-9; Social media usage: 1 = less than once a month, 2 = several times a month, 3 = once a week, 4 = 1–3 times a day, 5 = more than 3 times a day

Among the top 5 factors interfering with the use of online services were technical problems with Zoom or smartphone (n = 52, 24.4%) followed by internet instability (n = 40, 18.8%) and low concentration or poor memory (n = 33, 15.5%)(Table 7). Forty participants thought online health sessions did not deliver timely and individualized responses and guidance when compared with face-to-face sessions, and 15 participants (7.0%) faced a lack of concentration during online services. However, 15 participants (7.0%) felt online sessions were convenient and helped save time.

**Table 7:** Top things interfering online intervention and opinions of online intervention compared with face-to-face intervention (n = 213)

Rank	Things interfering with online intervention	Number	Opinions of online intervention	Number
1	Technical problems with Zoom or smartphone	52	No timely and individual response and guidance	40
2	Internet instability	40	Save time and convenient	26
3	Not concentrate or poor memory	33	Not concentrate	15
4	Environmental disturbance	32	Worse learning atmosphere	11
5	No timely and individual response and guidance	13	Content not clear	8

## Discussion

In this primary care programme in Hong Kong, 66.1% of older adults with multimorbidity used social media every day. Older adults preferred face-to-face services more than online services. Participants who declined to use online services were older, lived alone, had a lower income level, were more supported by social security, had greater cognitive decline, and were less depressed. Participants who did not respond to the feedback survey had fewer years of education and greater cognitive function. A higher level of education, internet connection issues, and lower level of self-efficacy on mobile apps were associated with less satisfaction with online services. Internet connection issues and a lower level of self-efficacy on mobile apps were associated with reduced preference for online services.

In this study, more than half of the participants used social media at least once every day, and almost 80% used social media at least once a month. Compared with older adults in other countries, the participants in this study were more tech-savvy regarding the use of social media. Among older adults aged 65 and above in the USA, only 45% reported they had ever used any social media sites in 2021 [32], and 42% reported owning smartphones [33]. Among UK older adults aged 60 years and above, 58% adopted smartphones in 2018, with the number decreasing with the increasing age [34]. The smartphone penetration in Asian older adults was 9.8% in China in 2018 and 10.6% in Japan in 2013 [35]. It was suggested that even though older adults adopted smartphones and had access to the internet, their online skills, such as social media skills, may vary greatly [36]. The usage of social media was high in Hong Kong and can make a foundation for the usage of online services in this aged population.

The current study found it feasible to provide online services to older adults in primary care in Hong Kong. Consistent with their social media use, the participation rate of online sessions was up to around 85%. Furthermore, the participants were overall satisfied with the online services. Against this background, inequalities in relation to skills and participation still exist that some older adults may not be able to or do not have the ability to receive online services [22]. When providing online services in the community or primary care settings, additional attention should be given to those who are older, live alone, are of lower income, receive greater social security assistance, and have greater cognitive decline.

Older adults may face some difficulties when using online services, which may also prevent them from participating. Internet connection issues were found to be a significant barrier to their satisfaction and preference for online services in this study. Strategies to promote participation and reduce barriers should be developed. The most fundamental is additional support and training to increase skills in using digital devices, connection, and using the internet [21]. Although related services shall be provided by educational services or other providers, healthcare workers can provide tailored necessary training prior to service commencement, which can be adapted according to the learning capability of older adults [21, 37]. Meanwhile, the design of online services can consider the specific needs of older adults regarding their different chronic conditions [38] and other needs such as timely and individual response and guidance, recording and playback. Self-efficacy can be another important factor to be enhanced when designing and providing such services, which is consistent with The Technology Acceptance Model (TAM) and how perceived usefulness, ease of use and behavioural intentions can impact actual use [7].

This study also had several limitations to acknowledge. First, the online services were offered due to the COVID-19 epidemic situation, which meant the participants could not choose the form of services. Second, the response rate of the feedback survey was only more than half, even after the research team called the non-responders once. Thirdly, we did not measure the efficacy of the online services. Digital inequalities may also arise in relation to efficacy. In future research, evidence on the effect of online interventions and their comparison with face-to-face interventions on health outcomes among primary care older adults are needed.

## Conclusion

More than half of older adults with multimorbidity used social media daily in Hong Kong primary care. Online services are feasible in primary care older adults in Hong Kong. Internet connection issues can be a significant barrier to the usage of online services in this population. Prior training and use of social media can be beneficial in the use and satisfaction of online services in older adults. Future studies are needed to explore the efficacy of online interventions on health outcomes among older adults in primary care.

## Data Availability

The dataset supporting the conclusions of this article is available from the corresponding author CKMW on request.
